# Effects of desensitizing treatments on acid-etched dentin: debris precipitation, bond strength and tag formation

**DOI:** 10.1007/s10266-026-01334-2

**Published:** 2026-03-11

**Authors:** Vitória Lacerda Santos, Alejandro Nicolás Tejada Medina, Antônia Patricia Oliveira Barros, Wilfredo Gustavo Escalante-Otárola, Cristiane de Melo Alencar, Milton Carlos Kuga

**Affiliations:** 1 Department of Restorative Dentistry, Araraquara Dental School, Sao Paulo State University—UNESP, Araraquara, SP Brazil; 2 School of Dentistry, Universidad Católica de Santa Maria - UCSM, Arequipa, Peru; 3 School of Dentistry, University Center of the State of Pará—CESUPA, Belém, PA Brazil

**Keywords:** Dentin sensitivity, Tooth erosion, Dentin desensitizing agents, Dentin permeability, Dentin bonding agents

## Abstract

To evaluate the effects of formulations containing potassium nitrate, silicate, and calcium phosphate, subjected to an acid challenge, on debris precipitation, quantification of exposed dentinal tubules, bond strength, failure mode, and tag formation. One hundred and twenty bovine incisors were randomly allocated into four groups: Desensibilize NanoP, Desensibilize KF 2%, Regenerate NR-5, and a control group. Specimens were immersed daily for 5 min in orange juice (pH 3.80 ± 0.04) and then rinsed with distilled water. Forty teeth were analyzed by scanning electron microscopy to assess debris precipitation and tubular occlusion. Another forty were subjected to micro-shear bond strength testing, and the remaining forty were analyzed under optical microscopy for tag formation. Data were analyzed using the Kruskal–Wallis/Dunn test (debris precipitation) and ANOVA/Tukey test (open tubules, bond strength, and tag formation), with a significance level of *α* = 0.05. The Regenerate group showed significantly higher debris precipitation, fewer open dentinal tubules, greater bond strength, and a higher incidence of cohesive failures. Tag formation was similar across all groups. The Regenerate protocol demonstrated superior performance in debris precipitation, tubule occlusion, bond strength, and tag formation, particularly when used in combination with a universal adhesive.

## Introduction

Dentin hypersensitivity (DH) is characterized as a sharp and transient pain resulting from the exposure of dentin to thermal, tactile, osmotic, or chemical stimuli. The occurrence of DH presupposes the exposure of dentin to the oral environment [1–5]. From a macroscopic point of view, sensitive and insensitive dentin appear similar; however, through scanning electron microscopy analysis, it is observed that, in hypersensitive areas, the openings of the dentinal tubules are wider, whereas, in non-sensitive regions, the tubule entrances are often occluded by hydroxyapatite crystals [2–4].

Among the etiological factors that favor dentin exposure, the loss of enamel tissue and the integrity of collagen stand out, which may occur as a result of erosion or abrasion processes. The synergistic interaction between these mechanisms enhances tooth wear and promotes the exposure of dentinal tubules [1, 2, 6]. Currently, erosion is considered the main agent responsible for the loss of dental structure, being defined as the dissolution of the dental surface by acids of non-bacterial origin. The acids involved in the erosive process can be classified as extrinsic or intrinsic. Exposure to extrinsic acids is commonly related to the consumption of acidic foods and beverages, such as citrus fruits and soft drinks, among others [7, 8].

In this context, there are two main therapeutic approaches to alleviate DH: one directed towards the blockage and obliteration of dentinal tubules, and the other interferes with neural transmission and interrupts the neural response to pain stimuli. Several materials and methods have been described in the literature for the treatment of DH, with therapies aimed both at the mechanical and chemical protection of the dentin-pulp complex and at the suppression of neural stimulation, always seeking to control the predisposing factors as well [3–5]. However, to date, no treatment that can be considered ideal, capable of eliminating the perception of pain in a predictable and permanent way, especially in the long term, has been identified.

The therapeutic strategies aim to reduce dentin permeability and reinforce the tissue’s resistance against chemical aggressions [3]. Among the proposed agents, the following stand out: Desensibilize KF2%^®^ (FGM Produtos Odontológicos, Joinville, SC, BR), composed of potassium nitrate, the NR-5 Serum Regenerate^®^ (Unilever, Compiègne, FR), containing calcium silicate, and the Nano-P Desensitizer^®^ (FGM Produtos Odontológicos, Joinville, SC), formulated with calcium phosphate nanoparticles, all indicated for the protection of cervical dentin. These formulations promote mineral precipitation on the dentin surface, in order to obstruct the tubules and form a protective layer resistant to acid challenges [4, 5, 9].

Although the desensitizing agents mentioned show promising potential in protecting exposed dentin, their effectiveness under conditions that simulate the oral environment, particularly in acidic diet scenarios, still requires further investigation. In addition to the ability to promote tubule sealing, it is essential that these agents do not compromise bonding to the dentin substrate, especially when universal adhesive systems are used, as bond strength is a critical parameter for clinical success.

In parallel with desensitizing approaches, universal adhesive systems have gained recognition for their versatility and ability to achieve effective bonding to dentin substrates [10, 11]. These adhesives, formulated with the functional monomer 10-MDP, can be applied using either the self-etch or the total-etch technique, providing a stable and durable adhesive interface. The 10-MDP chemically interacts with dentin, promoting the formation of a thick and stable hybrid layer, with satisfactory clinical performance regardless of the technique employed [12–14].

Therefore, the aim of this study was to evaluate the effects of applying formulations containing potassium nitrate (Desensibilize KF 2%), silicate (Regenerate), and calcium phosphate (Desensibilize Nano-P), subjected to a simulated acidic challenge, by analyzing debris precipitation, quantification of exposed dentinal tubules on the cervical root surface, bond strength, failure mode, and tag formation. The null hypothesis was that no significant differences would be observed among the treatment protocols for any evaluated parameter, regardless of acidic challenge.

## Materials and methods

One hundred twenty sound bovine incisors were stored in 0.1% thymol solution at 4 °C for seven days.

### Treatment protocols

The specimens were divided into four groups according to the treatment protocol (*n* = 120). Forty teeth were used for the analysis of residue precipitation and dentinal tubule occlusion (*n* = 10, each), forty teeth for the evaluation of bond strength by micro-shear testing (*n* = 10, each), and another forty teeth for investigating tag formation (*n* = 10, each).

**DKF** (Desensibilize KF2%; FGM, Joinville, SC, BR): 5% potassium nitrate associated with 2% sodium fluoride: Gel was applied on dentin substrate, left untouched for 10 min and then removed using 10 mL water distilled. A total of 4 applications were performed with a seven-day interval between sessions [15];

**RGN** (NR-5 Serum Regenerate; Unilever, Compiègne, FR): Calcium silicate and sodium monofluorophosphate associated with sodium fluoride (Activator Gel Regenerate; Unilever, Olgiate Comasco, IT). Firstly, a thin layer of NR-5 Serum Regenerate gel was applied on dentin substrate. Then, Activator Gel was applied over that layer and mixed for 5 s using a plastic spatula. That resulting material was kept on dentin for 3 min and removed using 10 mL distilled water. A total of 3 consecutive applications were performed, with a 24-h interval between sessions, according to the manufacturer's instructions. After the fourth day, daily brushing using electric tooth brush (Care 500; Procter & Gamble, São Paulo, SP, BR) and dental gel (Advanced Toothpaste Regenerate; Unilever, Compiègne, FR) were carried out for 10 s, for 30 days;

**DSN** (Desensibilize Nano P; FGM, Joinville, SC, BR): Calcium phosphate nanoparticles associated with 5% potassium nitrate and sodium fluoride- 9000 ppm gel was directly applied on dentin and rubbed using a rubber cup (X-Smart Plus; Dentsply, York, PE, USA) for 10 s at 200 rpm and rested for 5 min. The gel was removed using 10 ml distilled water. A total of 4 applications were performed, with 7-day intervals between them.

**CRT** (control): No chemical agent, material, or additional procedure was applied, allowing evaluation of the dentin substrate without interference from prior treatments.

Between each session, the specimens were stored in artificial saliva (CaCl_2_.2H_2_O, 0.167 g; KH_2_PO_4_, 0.123 g; KCl, 11.2 g; (HOCH_2_)3CNH_2_, 2.42 g; distilled water), pH 7.0 at 37° C. The specimens were subjected to acid challenge were daily immersed for 5 min in orange juice (Minute Mais; Coca Cola, Araraquara, SP, BR), pH 3.80 ± 0.04, and then rinsed using distilled water [16, 17]. Table 1 shows the specifications of the materials used in this study.

**Table 1:** Show commercial names, composition and manufacturer of the experimental products used in study

Group	Composition	Manufacturer
DKF—Desensitize KF 2%	KNO3 (5%) + NaF (2%)	FGM, Brazil
RGN—Regenerate NR-5	Glycerin, Calcium silicate, PEG-8, Hydrated silica, Trisodium phosphate, Sodium phosphate, Aqua, PEG-60, Sodium lauryl sulfate, Sodium monofluorophosphate, Synthetic fluorphlogopite, Sodium saccharin, Polyacrylic acid, Tin oxide, limonene, CI 77891. 1450 ppm fluoride	Unilever, Kingdom, United
DSN—Desensitize NanoP	Calcium phosphate (nano-HAP), Potassium nitrate and 9000 ppm sodium fluoride	FGM, Brazil
CRT—Controle negativo	–	–
Potassium nitrate; calcium silicate, calcium phosphate		

### Debris precipitation and open dentinal tubules

#### Specimens’ preparation

Fragments (10 mm length × 10 mm width x 5 mm thickness) were obtained from the tooth middle third using a hard tissue cutting machine (Isomet 100, Buehler, Lake Bluff, IL, USA) under cooling running water. The fragments´ buccal surfaces were flattened in a polishing machine (DP-10; Panambra, Struers, Ballerup, DI) using #600, #1200 and #1500 sandpapers for 20 s (ISO 6344–1), for the dentin exposure and planning in order to standardize the smear layer. The specimens were previously immersed in 0.6% hydrochloric acid solution at 37° C for 5 min. Afterwards, the specimens were rinsed using distilled water and stored in artificial saliva until ready to use.

#### Scanning electron microscopy analysis

After the desensitization protocols, the specimens were kept in artificial saliva for 24 h. In sequence, they were dried inside a closed chamber containing colloidal silica for 7 days. Then, the specimens were mounted onto metal stubs, gold-sputter coated (single cycle-120 s) under vacuum, inside a metalizing chamber (MED 010, Balzers Union, Balzers, Listenstain) and examined using scanning electron microscopy (JEOL 6060; JEOL Ltda, Tokyo, Japan) operating at 20 kV. All images were obtained by only one operator.

Representative image was obtained at 500 × magnification for debris precipitation evaluation on dentin. Other two calibrated and independent examiners classified the debris presence according to Kuga et al. [18]:

Score 1: Absence or discrete debris precipitation, with visible dentinal tubules opening;

Score 2: Discrete debris precipitation, with the precipitation of residues on the dentin occupying up to 50% of the dentin surface;

Score 3: Moderate debris precipitation, with the precipitation of residues on the dentin occupying between 50% and 75% of the dentin surface;

Score 4: Intense debris precipitation, with the precipitation of residues on the dentin occupying more than 75% or all of the dentin surface

Another image at 2000 × magnification was obtained from the central region of the middle dental third of all specimens to count the unblocked and open dentinal tubules. Two different examiners counted the open dentinal tubules in each image (Kappa score ≥ 0.8) of the specimens using Photoshop CS6 program. The results obtained by the two examiners were used to calculate the mean value that was determined for the specimen.

### Micro-shear bond strength (µSBT)

#### Specimens’ preparation

Forty incisor bovine crowns were sectioned in mesio-distal direction using the a low-speed precision cutting machine under constant water irrigation and new fragments (10 mm length × 5 mm width) were acquired. The buccal cervical surface fragment was flattened with a polishing device (DP-10; Panambra, Struers, Ballerup, DI) using #180 SiC paper to obtain planning and dentinal tubules exposure.

Subsequently, the exposed dentin surface was polished following the sequence #320 and #600 of SiC papers on each fragment until its surface was adequate polished. Fragments were placed into a polystyrene matrix mold (16.5 mm width × 25.0 mm length) and embedded with acrylic resin (Classic Jet, São Paulo, SP, Brazil) and left undisturbed for 24 h. After inclusion inside the matrix, specimens were randomly divided into four groups (*n* = 10) and submitted to the treatment protocols and acid challenge treatment, in accordance to previously described (item 2.1).

#### Bonding procedures

The bonding procedures were conducted with an universal adhesive (Scotchbond Universal; 3 M ESPE, St Paul, MN, USA), in self-etching strategy, actively applied for 20 s using a microbrush (Cavibrush; FGM, Joinville, SC, Brazil), dried with an oil-free air-spray for 5 s, and light-cured for 20 s with 1000 mW/cm^2^ (Quazar; UFGM, Joinville, SC, BR), in all protocols.

After the bonding procedures were performed, four cylinders made of composite resin (Filtek Z-350; 3 M ESPE, St Paul, MN, USA) were bonded on the buccal surface. Two of those were placed at mesial and two were placed at distal side and light-cured for 20 s with 1000 mW/cm2. A transparent tygon tube matrix (Tygon tube, R-3603, Saint-Gobain Performance Plastics, Maime Lakes, FL, USA) with 0.7 mm internal diameter and 1.0 mm height was used for the composite resin filling cylinders on the specimen’s surface.

#### Bond strength test

In the sequence, all the specimens were stored in a 95% relative humid environment, int 37 ºC, and µSBT was performed after 24 h. All the fragments with the composite cylinders were fixed inside the metal matrix and those cylinders were then placed perpendicularly to a 500 N cell. An orthodontic wire (0.2 mm diameter) held the composite cylinder base and all the specimens were subjected to tensile loading with 0.5 mm/min crosshead speed using an universal electromechanical testing machine (OM150; Odeme, Luzerna, SC, Brazil) until composite cylinder specimen’s full displacement.

The bond strength was obtained from maximum force (N) divided by union area (mm^2^), in MPa. The arithmetic average was calculated for the four composite cylinders from each fragment and called specimen´s mean.

### Failure mode

Failure mode was subjected to optical microscope analysis at 20 × magnification (Alliance, São Carlos, SP, Brazil), and classified as: adhesive failure, when occurred at interface adhesive layer between dentin and composite resin; cohesive failure, within composite resin or in dentin; and mixed failure, when both types of failure were combined.

### Extension tags formation

#### Specimens’ preparation

Forty crowns were cross sectioned in cervical level using a diamond disc at low speed (Isomet; Buehler Ltd., Lake Bluff, IL, USA). The buccal cervical face was flattened with #180 silicon carbide sandpaper (Norton, São Paulo, SP, Brazil) under distilled water refrigeration in the circular polisher (Panambra, Struers, Ballerup, Denmark). The exposed dentine was polished using #320 and #600 sandpapers, for 20 s under distilled water refrigeration.

The specimens were divided in 4 groups (*n* = 10, each) and submitted to treatment protocols as described in item 2.2.

#### Restorative procedures

Initially a universal adhesive (Scotchbond Universal; 3 M ESPE, St Paul, MN, USA), was applied, as described in item 2.3.1. Rhodamine B (Synth, São Paulo, Brazil) was incorporated in universal adhesive to extension tags formation analysis. In sequence, the specimens were restored with three increments, each 1 mm thick, using composite resin (Filtek Z-250, 3 M, St. Paul, MN, USA) and submitted to photoactivation with an LED device (Quazar; UFGM, Joinville, SC, BR), for 40 s, in 1000 m/W cm^2^, after placing each increment.

At the end, a specimen with approximately 3 mm thickness of composite resin on the buccal cervical surface of the dental crown was obtained. In sequence, the crowns were longitudinally sectioned in the buccolingual direction using a hard tissue cutting machine (Isomet, Buehler). The distal section of each specimen was selected, and the dentin-restoration interface was gradually ground with #600 and #1200 silicon carbide abrasive papers (Norton), under cooling with distilled water, using a circular polisher (Arotec, Cotia, SP, Brazil).

The specimens were then washed with distilled water, and the ground surface was gradually polished with aluminum oxide at grit sizes of 30, 10, and 5 μm, and a felt disc, again using the circular polisher. Once the surface polishing was completed, the specimens were immersed in distilled water and subjected to agitation in an ultrasonic chamber (Cristófoli, Campo Mourão, Brazil) for 5 min.

### Confocal analysis

An image of each section was obtained using confocal laser microscopy (LSM 800 Airyscan, Carl Zeiss, Oberkochen, Germany) with a magnification of 20 × . The absorption and emission wavelengths for Rhodamine B were 540 nm and 494 nm, respectively.

Ten measurements (in micrometers) of the greatest extension of tags formed in the dentin were obtained using Image J software (National Institutes of Health, Bethesda, MD, USA), and the arithmetic mean of these measurements was defined as the extension tags formation for the specimen under analysis.

### Statistical analysis

The data were initially subjected to the Shapiro–Wilk test to assess normality. After confirmation of a non-normal distribution, the data related to debris precipitation were analyzed using the nonparametric Kruskal–Wallis test, followed by Dunn’s post hoc test. In contrast, the data for open dentinal tubule counts, microshear bond strength, and tag formation showed a normal distribution and were therefore analyzed using ANOVA, followed by Tukey’s post hoc test. All statistical analyses were performed at a significance level of 5% (*α* = 0.05).

## Results

### Debris precipitation and open dentinal tubules

**Fig. 1:**
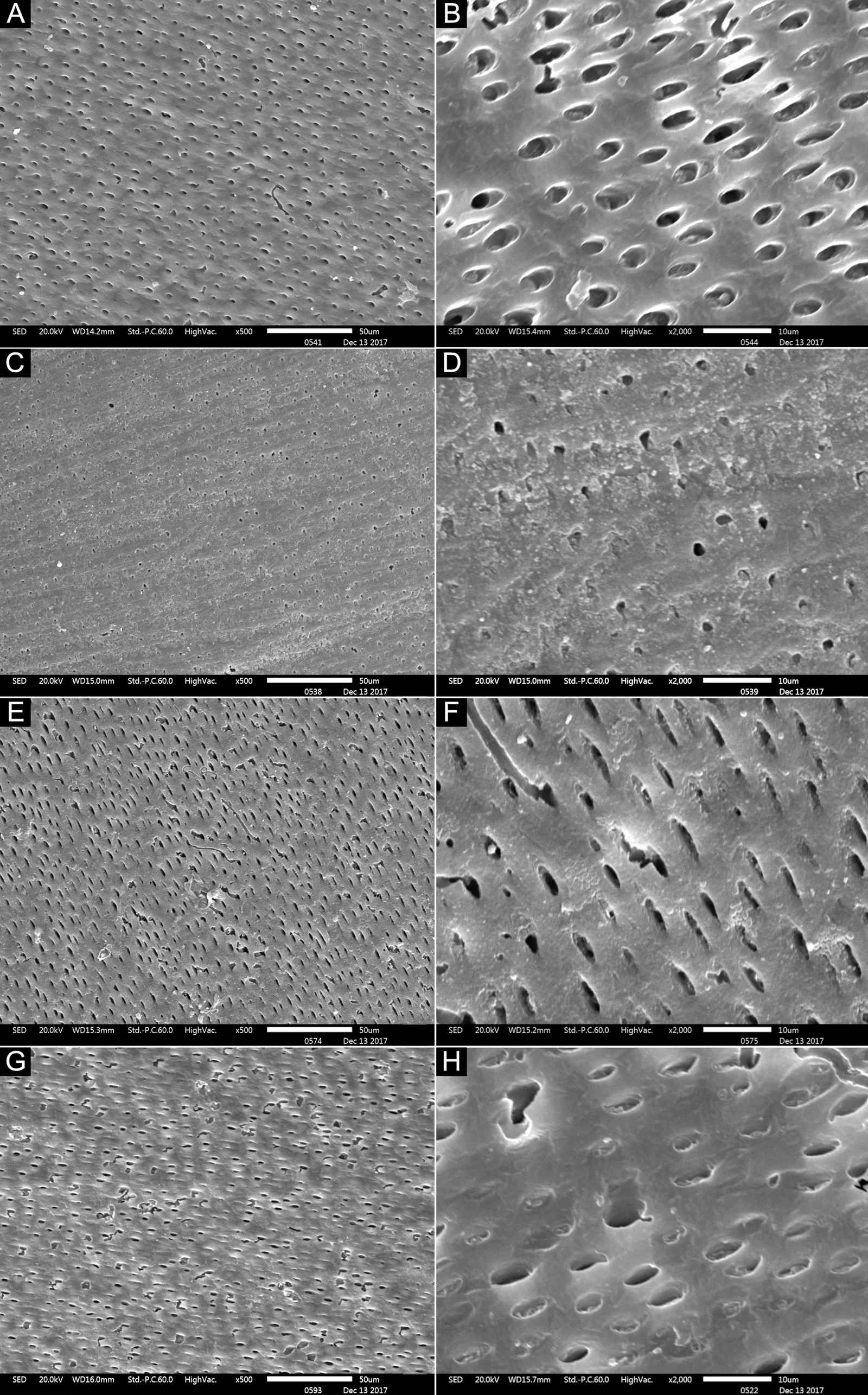
Show the debris precipitation pattern on dentin and the dentinal tubules amount after different dentin desensitization protocols, with acid challenge. DKF, Desensibilize KF 2%; (**A** and **B**); RGN, NR-5 Serum Regenerate; (**C** and **D**); DSN, Desensibilize Nano P (**E** and **F**); CRT, control; (**G** and **H**)

Regarding debris precipitation on the cervical dentin surface, regardless of the acid challenge, RGN showed the highest debris precipitation among the other groups (*P* < 0.05; Fig. 1 and Table 2), which were similar to each other (*P* > 0.05; Fig. 1 and Table 2).

**Table 2:** Median, minimum and maximum values, first and third quartile of debris precipitation, and open dentinal tubules amount arithmetic mean and standard deviation on dentin surface, after different treatment protocols without acid challenge

		DKF	RGN	DSN	CRT
Debris	median	1^b^	4^a^	1^b^	1^b^
	min/max	1–2	3–4	1–2	1–1
	1Q/3Q	1.0–1.0	4.0–4.0	1.0–1.5	1.0–1.0
Open dentinal tubules	Mean (sd)	20.71 (4.00)^b^	6.30 (1.77)^a^	19.50 (2.95)^b^	20.40 (2.62)^b^
	CI	18.22 < µ < 23.18	5.21 < µ < 7.41	17.67 < µ < 21.33	18.78 < µ < 22.02

^ab^Different letters at the same row indicate significant difference (*P* < 0.05)

*1Q and 3Q* first and third quartiles, *min* minimum value, *max* maximum value, *sd* standard deviation, *CI* confidence interval, *DKF* Desensibilize KF 2%, *RGN* NR-5 Serum Regenerate, *DSN* Desensibilize Nano P, *CRT* control

On the other hand, RGN presented the lowest open dentinal tubules incidence (*P* < 0.05; Fig. 1 and Table 2), but the other groups showed similar open dentinal tubules amount (*P* > 0.05; Fig. 1 and Table 2).

### Micro-shear bond strength (µSBT)

RGN showed the highest bond strength (*P* < 0.05; Table 3). There were no differences among other protocols (*P* > 0.05; Table 3).

**Table 3:** Arithmetic media, standard deviation and confidence interval of micro-shear bond strength (in MPa) and extension tags (in µm) formation in dentin substrate, in function of desensitization protocols

		DKF	RGN	DSN	CRT
BS	m (sd)	22.61 (2.54)^b^	28.69 (2.53)	25.29 (3.11)	23.29 (2.69)
	CI	21.03 < µ < 25.15	27.12 < µ < 30.26	23.36 < µ < 27.22	21.63 < µ < 24.96
ET	m (sd)	163.62 (17.42)^a^	170.04 (13.54)a	165.64 (14.97)^a^	165.42 (8.32)
	CI	152.82 < µ < 174.42	161.65 < µ < 178.43	156.36 < µ < 174.91	160.26 < µ < 170.58

^ab^Different letters in same row show statistical differences (*P* < 0.05)

*BS* micro-shear bond strength, *ET* extension tags formation, *m* media, *sd* standard deviation, CI confidence interval, *DKF* Desensibilize KF 2%, *RGN* NR-5 Serum Regenerate, *DSN* Desensibilize Nano P, *CRT* control

### Failure mode

**Fig. 2:**
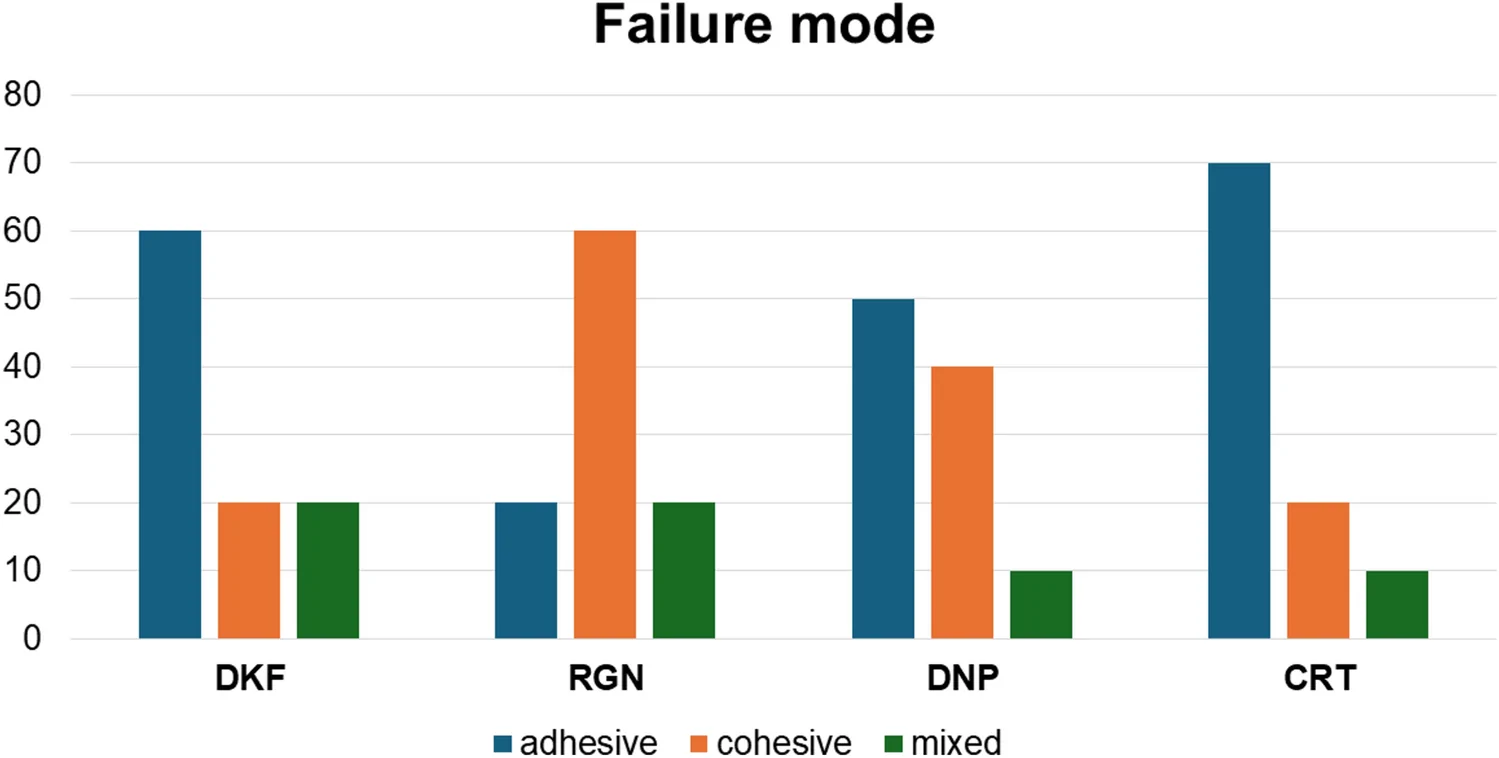
Adhesive failure mode as a function of the desensitization protocol applied to the dentin substrate. *DKF* Desensibilize KF 2%, *RGN* NR-5 Serum Regenerate, *DNP* Desensibilize Nano P, *CRT* Control

RGN showed a higher incidence of cohesive failures, whereas the other protocols showed a higher incidence of adhesive failures. Figure 2 show failure mode incidence of the universal adhesive in the adhesive interface, in function of the desensitization protocol applied on dentin substrate.

### Extension tags formation

**Fig. 3:**
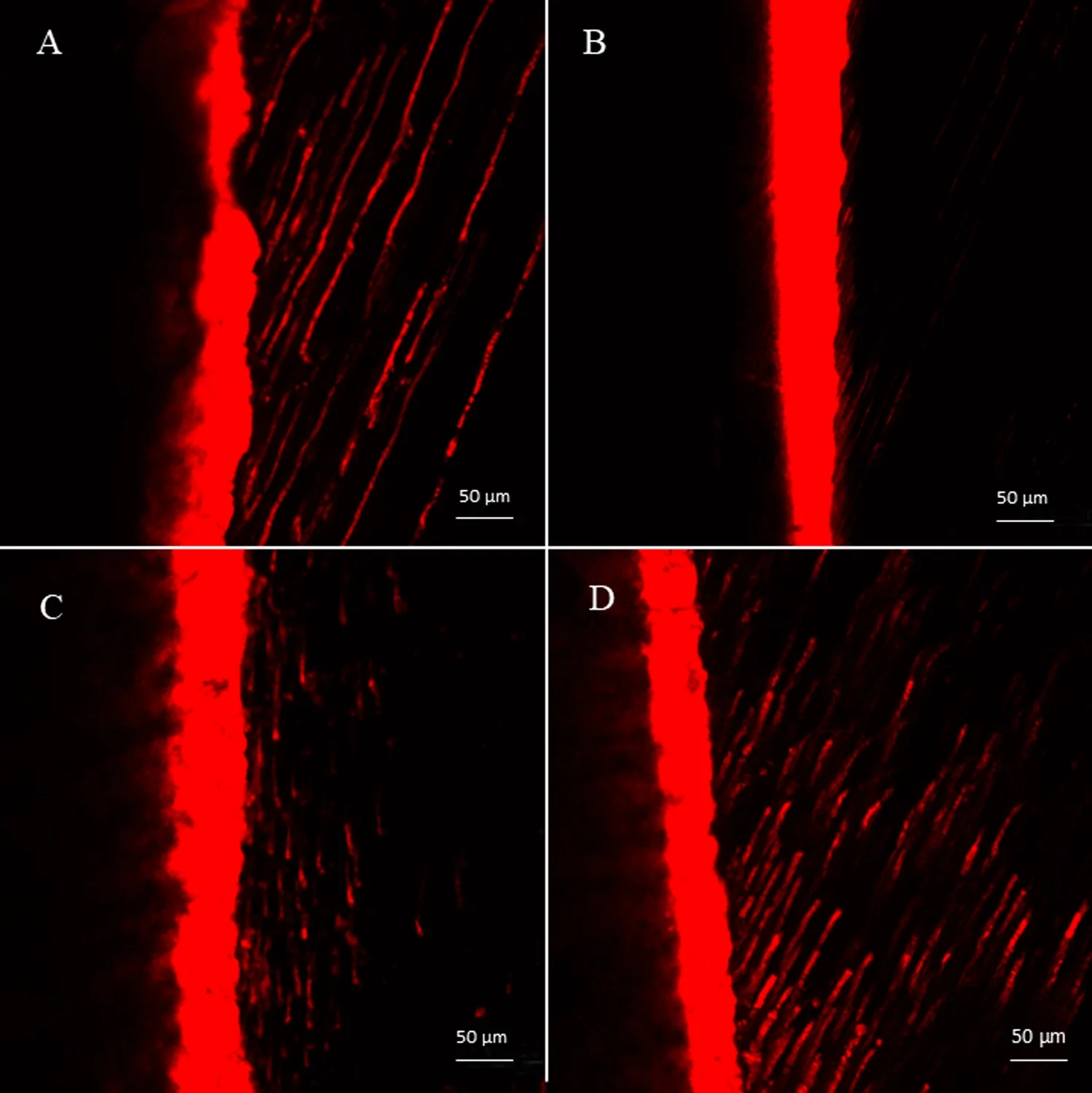
Extension tags formation (in mµ) representative confocal laser images, in function of desensitized protocols. *DKF* Desensibilize KF 2% (A) control (B), *DSN* Desensibilize Nano P (C), *RGN* NR-5 Serum Regenerate (D). Scale: 50 µm

All protocols showed similar extension tags formation in dentin substrate (*P* > 0.05, Fig. 3 and Table 3).

## Discussion

This study evaluated the effects of applying formulations containing potassium nitrate, silicate, and calcium phosphate subjected to a simulated acid challenge, through the analysis of debris precipitation, quantification of exposed dentinal tubules on the cervical root surface, bond strength, failure mode, and tag formation. The treatment protocol using the calcium silicate–based formulation (RGN) showed statistically significant differences (*P* < 0.05) compared with the other groups, exhibiting greater debris precipitation, reduced dentinal tubule exposure, and higher bond strength. Therefore, the null hypothesis was rejected.

Bovine dentin was used in this study because it presents chemical composition, tubular morphology, and mechanical behavior similar to those of human dentin [19]. Furthermore, the acid challenge protocol adopted [16, 17] was designed to simulate adverse clinical conditions frequently observed in the oral environment, such as pH fluctuations associated with an acidic diet or erosive processes.

RGN is a gel composed of calcium silicate salts and sodium phosphate (monosodium phosphate and trisodium phosphate), combined with 1450 ppm of fluoride, in the form of sodium monofluorophosphate, and is commonly used as an adjunct in the prevention and treatment of enamel erosion [20]. When in contact with the moist environment, the silicate promotes the formation of a hydroxyapatite layer on the dentin surface, resulting in the obstruction of the tubules and increased tissue resistance to acid challenges [20].

In our study, it was found that mineral precipitation occurred in the form of a homogeneous debris layer on the cervical root dentin surface, which causes significant obstruction of the dentinal tubules, regardless of exposure to the acid challenge. Additionally, the daily application of the RGN gel may have favored the intensification of the formation of this mineralized layer, thus contributing to the superiority observed compared to the other treatment protocols.

Nano-hydroxyapatite (DSN) acts predominantly by obliterating dentinal tubules through both chemical and physical mechanisms [21]. Chemically, the presence of 9000 ppm of fluoride inhibits cariogenic bacterial activity, promoting remineralization and desensitization, while 5% potassium nitrate depolarizes nerve fibers, contributing to the relief of hypersensitivity [21]. Physically, the nanometric size of hydroxyapatite particles facilitates their penetration into dentinal tubules and enamel microcracks, ensuring effective sealing and aiding in the restoration of the tooth’s microstructure and remineralization [4]. The product also exhibits appropriate wettability and solubility properties, enabling the controlled release of calcium and phosphate ions into the oral environment [22].

However, in our study, when subjected to a daily acid challenge with citric acid (orange juice) [15], it was observed that DSN resulted in a lower number of open dentinal tubules compared to DKF, despite debris precipitation being similar on the dentin surface. These results suggest that the debris generated by the chemical components of DSN, especially in the intertubular dentin, are more soluble to citric acid than those formed by RGN. However, the obstruction of the dentinal tubule openings was more effective with DSN, which corroborates the clinical results observed by Wang et al. [23] in the treatment of dentin hypersensitivity.

In an in vitro study, Escalante-Otárola et al. [24] evaluated the efficacy of different desensitizing agents, including 2% sodium fluoride (DKF). Using one hundred and fifty bovine incisors with preserved crowns and root anatomies, the authors observed that DKF presented the lowest bond strength value among the materials tested. These findings are consistent with the results obtained in our study, which also demonstrated the lower effectiveness of DKF (*P* > 0.05) in maintaining bond strength.

In the failure mode analysis, our results showed that the protocol with the RGN gel had a higher incidence of cohesive failures, while the other protocols showed a predominance of adhesive failures. These findings suggest that treatment with RGN favors a more robust interaction between the adhesive and the dentin substrate, likely due to the formation of a more stable hydroxyapatite layer resistant to demineralization, in agreement with the results of previous studies [25–27].

Comparing all the protocols tested for the formation of extension tags on the dentin substrate, no statistically significant differences were found (*P* > 0.05). In other words, all products were effective regardless of the mechanism of action. Therefore, these findings suggest that, regardless of the desensitizing protocol applied, there is a uniform action in the penetration of the adhesive into the microstructures of the dentin [28–30].

The performance observed for RGN may be related to the use of a universal adhesive system containing the functional monomer 10-MDP, which is capable of promoting partial dentin demineralization and forming a thin hybrid layer (less than 1 µm), with high chemical affinity for the calcium ions in hydroxyapatite present in dentin tissue [31, 32]. This interaction favors the formation of a stable adhesive interface, contributing to higher bond strength, even in substrates previously treated with desensitizing agents. Thus, the effectiveness in tag formation may reflect the ability of this adhesive to interact with different dentin modifications induced by the applied agents, ensuring proper infiltration into dentinal microstructures [32, 33]. Fehrenbach et al. [34], through a meta-analysis of in vitro studies, demonstrated that self-etch adhesives containing 10-MDP exhibited greater bond strength to dentin. The authors attributed this performance to the stable chemical interaction of 10-MDP with hydroxyapatite, reinforcing its effectiveness in the durability of the adhesive interface and its relevance in the formulation of universal adhesives, corroborating findings from other studies.

From a clinical perspective, the results obtained in this study indicate that the use of the RGN gel may represent a particularly advantageous strategy in situations of dentin exposure, especially in contexts involving subsequent adhesive procedures. This finding is supported by a randomized controlled clinical study conducted by Fernandes et al. [35], which evaluated 48 volunteers diagnosed with dentin hypersensitivity, who were allocated into two groups: one treated with a bioactive calcium silicate-based dental gel and another control group. The results showed that the protocol containing silicon and calcium was effective in the immediate reduction of pain associated with dentin hypersensitivity, suggesting that the combination of a bioactive gel enriched with calcium silicate constitutes a promising therapeutic approach in managing this condition.

The results of this study provided valuable contributions to understanding the efficacy of different desensitizing agents in challenging conditions. Despite strict experimental control, the model cannot fully reproduce the complexity of the oral environment, including saliva, biofilm, and dynamic pH variations [36, 37]. The results reflect only immediate adhesive performance, and factors such as hydrolytic degradation, enzymatic activity, and mechanical fatigue may compromise long-term stability. Caution is advised when extrapolating these findings, and studies with long-term aging and human teeth are recommended.

Finally, the present study demonstrated that the Regenerate protocol showed superior performance in debris precipitation, tubular occlusion, bond strength, and tag formation, particularly when used in combination with a universal adhesive.

## Data Availability

None.
